# Subsidence prediction of overburden strata and ground surface in shallow coal seam mining

**DOI:** 10.1038/s41598-021-98520-9

**Published:** 2021-09-23

**Authors:** Jian Cao, Qingxiang Huang, Lingfei Guo

**Affiliations:** 1 Institute of Mining Engineering, Inner Mongolia University of Science and Technology, Baotou, 014010 Inner Mongolia China; 2 School of Energy, Xi’an University of Science and Technology, Xi’an, 710054 Shaanxi China

**Keywords:** Geodynamics, Civil engineering

## Abstract

Shallow coal seam with thick soil layer is widely reserved in the Jurassic Coalfield, Western China, mining-induced subsidence represents complex characteristics. Combining with physical simulation, theoretical analysis and in-situ observation, the overburden strata structure in dip direction were revealed, and the subsidence prediction models were established, based on this, the subsidence equations of overburden strata and ground surface were proposed. The results show that after shallow coal seam mining, based on the subsidence and movement characteristics, the overburden strata structure can be divided into three zones, which are "boundary pillar F-shape zone" (BPZ), "trapezoid goaf zone" (TGZ) and "coal pillar inverted trapezoidal zone" (CPZ). The subsidence of overburden strata depends on the key stratum, while the subsidence of soil layer depends on the bedrock subsidence basin, which is between the bedrock and thick soil layer. The bedrock subsidence is mainly related to mining height and bulking coefficient in TGZ, while it is mainly affected by mining height and distribution load on the key stratum in BPZ and CPZ. According to physical simulation and theoretical model, the maximum surface subsidence of No.1-2 seam mining in Ningtiaota coal mine are 1.1 m and 1.07 m respectively, which is basically consistence with the result of in-situ observation (1.2 m).

## Introduction

The Jurassic Coalfield in Shaanxi Province, Western China, mainly reserves shallow coal seam with thick soil layer. Recently, due to high-strength underground mining, the subsidence of ground surface is serious, which easily causes the decline of groundwater level and desertization^1–3^. Taking Hongjiannao Scenic Area in northern of Shaanxi as example, from 1992 to 2000, the water level drops about 1.1 m, and the lake area decreases from 55 to 48 km^2^, since 2000, the lake area decreases about 30%^4^. Besides, ground surface represents obvious uneven subsidence, subsidence of overburden strata and ground surface above goaf is larger, while it is smaller above pillar^5^, and the uneven subsidence has obvious effect on the surface buildings^6^. Therefore, under mining conditions of shallow coal seam with thick soil layer, how to realize subsidence prediction of overburden strata and ground surface, it is a necessary and complicated scientific problem to be solved.

Overburden strata in the Jurassic Coalfield has its own characteristics, overall, the thick soil layer is widespread above the bedrock^7^. Due to soil layer and bedrock belong to two different media, the subsidence characteristics are also different^8–10^. In order to obtain quantitative subsidence prediction method, it is of great significance to study further.

To date, in-situ observation was widely applied to study the mining-induced ground surface subsidence. Xu et al.^11^ obtained the surface dynamic movement parameters, and the subsidence velocity prediction equation of arbitrary point in advance profile was determined. Liu et al.^12^ divided the mining process into the initial mining stage, the normal periodic stage and the final mining stage, the surface movement of different stages was studied. Xu et al.^13^ analyzed the control effect of key strata on overburden and surface, and the effect of key block lumpiness on the subsidence curves was revealed. Zhu et al.^14^ found that the main key strata is the control layer of overburden strata and surface. Wu et al.^15^ revealed the control effect of thick and hard strata on the surface subsidence. Baek et al.^16^ studied the mining-induced ground subsidence in Korea by SAR interferometry. Using TimeSAR in Springfield, USA, Grzovic et al.^17^ evaluated the ground subsidence, and also measured the temporal pattern of deformation.

Furthermore, the other two methods, numerical simulation and physical simulation were also used to simulate the subsidence of overburden strata and surface. In order to observe the surface movement in thick loose layer high-intensity mining, Zhao et al.^18^ analysed the failure characteristics of overburden strata and surface subsidence. Wang et al.^19^ studied the subsidence and stress distribution of overburden strata, and it showed that there only exists caving zone and fracture zone in shallow coal seam mining. Xu et al.^20^ revealed the effect of primary key stratum on the dynamic surface subsidence. Liu et al.^21^ established the numerical model of strip-pillar mining, the surface subsidence and horizontal movement contours under different alluvium thickness were given. Wu et al.^22^ found that the subsidence of soil layer was closely related to its own property. Based on deep mining and shallow mining, Xu et al.^23^ studied the effect of key strata on ground surface subsidence. Taking three typical shallow coal seam conditions as the background, Fan et al.^24^ analyzed the movement and fractures of overburden strata in the horizontal and vertical direction. Based on the geological information gathered from the GIS and MIS, Unlu et al.^25^ established a number of two dimensional finite element model to analyse the ground subsidence occurring due to mining. Alejano et al.^26^ studied the FDM predictive methodology for subsidence in inclined seam mining.

In addition, theoretical analysis related to the subsidence of overburden strata and surface was studied. Yang^27^ put forward a prediction method of surface based on the boundary value method. Wang et al.^28,29^ found the relationship between mining degree and subsidence pattern by rock mechanics. Under the mining conditions of thick alluvial soil, Zhang et al.^30^ regarded the soil and bedrock as random medium and viscoelasticity beam respectively, and the calculation method of surface subsidence prediction was put forward. Wang et al.^31^ divided the strata movement into four stages, and the movement models of overburden strata were established. Hou^32^ analysed the effect of overburden property on the surface movement, it is found that the softer the overburden strata, the greater effect on the maximum surface subsidence values, and the greater the dip angle of the coal seam, the larger change of the maximum surface subsidence values. Based on pooling and meta-analysis of empirical data from a number of different countries and coalfields, McCay et al.^33^ proposed an universal tool for the estimation of maximum subsidence. Karmis et al.^34^ analysed the application of the influence function method for ground movement predictions, and demonstrated the applicability in U.S. coalfields. Singh et al.^35^ established a visco-elastic model to predict the mining-induced surface subsidence in Indian coalfields.

Besides, other studies were carried out to reveal the subsidence characteristics of overburden strata under back-filling mining^36–40^. However, the previous studies were largely limited to the subsidence of overburden strata and surface above goaf, quantitative subsidence above pillar still remains unclear. In addition, subsidence prediction method in shallow coal seam with thick soil layer is less studied. Therefore, based on typical mining conditions in Ningtiaota coal mine, combining with physical simulation, theoretical analysis and in-situ observation, we studied subsidence characteristics of overburden strata and ground surface, the subsidence prediction models were established, and the prediction method were put up. This study is conducted to provide a new subsidence prediction method in shallow coal seam mining.

## Subsidence law of overburden strata and ground surface

### Engineering background

**Figure 1:**
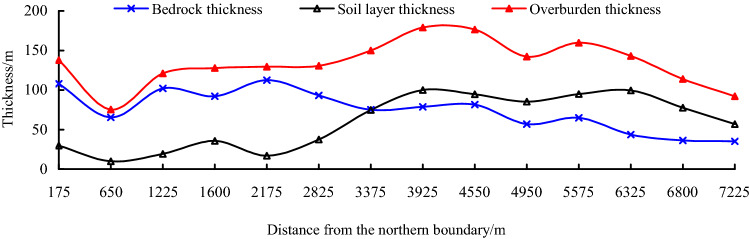
The thickness of bedrock and soil layer in Ningtiaota coal mine.

Ningtiaota coal mine is located in Shennan mining area, its mining conditions are mainly characterized by shallow depth (less than 200 m), nearly horizontal (1°–3°), and thick red soil is widespread over the bedrock. No. 1-2 and No. 2-2 seams are mainly mined, according to drillholes NBK8, NBK16, NBK22, NBK26 and NBK29, the thickness of bedrock and soil is shown in Fig. 1, overall, the average thickness of bedrock and soil are 74.65 m and 59.52 m respectively.

According to drillhole NBK26, the thickness of No. 1-2 seam is 1.9 m, and it mines 176.6 m deep, the bedrock thickness is 81.9 m, the soil thickness is 94.7 m, the width of coal pillar is 20 m, parameters of the coal, its roof and floor are listed in Table 1.

**Table 1:** The parameters of coal and its roof and floor.

Lithology	Thickness (m)	Depth (m)	Bulk density (kg/m^3^)	Compressive strength (MPa)	Cohesion (MPa)	Poisson's ratio
Red soil	94.70	94.70	1860	0.29	0.77	0.35
Sandymudstone	14.80	109.50	2560	34.70	1.15	0.24
Siltstone	21.60	131.10	2420	31.90	0.65	0.32
Medium-grained sandstone	28.80	159.90	2160	35.30	0.80	0.29
Siltstone	6.70	166.60	2420	31.90	0.65	0.32
Medium-grained sandstone	10.00	176.60	2330	40.60	1.50	0.28
No. 1-2 seam	1.90	178.50	1290	15.70	1.10	0.28
Fine-grained sandstone	9.40	187.90	2270	29.60	1.50	0.27
Siltstone	3.80	191.70	2440	36.00	0.90	0.30
Fine-grained sandstone	5.90	197.60	2340	48.50	1.90	0.29
Siltstone	1.00	198.60	2400	45.30	1.20	0.30
Fine-grained sandstone	13.20	211.80	2300	45.60	2.20	0.27
No. 2-2 seam	4.60	216.40	1340	13.80	1.20	0.27
Siltstone	3.50	219.90	2340	20.50	0.15	0.34
Fine-grained sandstone	8.70	228.60	2280	39.10	2.20	0.27
Siltstone	2.40	231.00	2400	42.50	0.70	0.31
Fine-grained sandstone	11.70	242.70	2350	47.50	2.40	0.27
Medium-grained sandstone	6.90	249.60	2260	41.90	2.50	0.26
Siltstone	3.50	253.10	2400	46.30	1.80	0.28
No. 3-1 seam	2.70	255.80	1270	10.90	1.10	0.29
Fine-grained sandstone	2.00	257.80	2330	43.10	2.00	0.25

### Physical simulation

**Figure 2:**
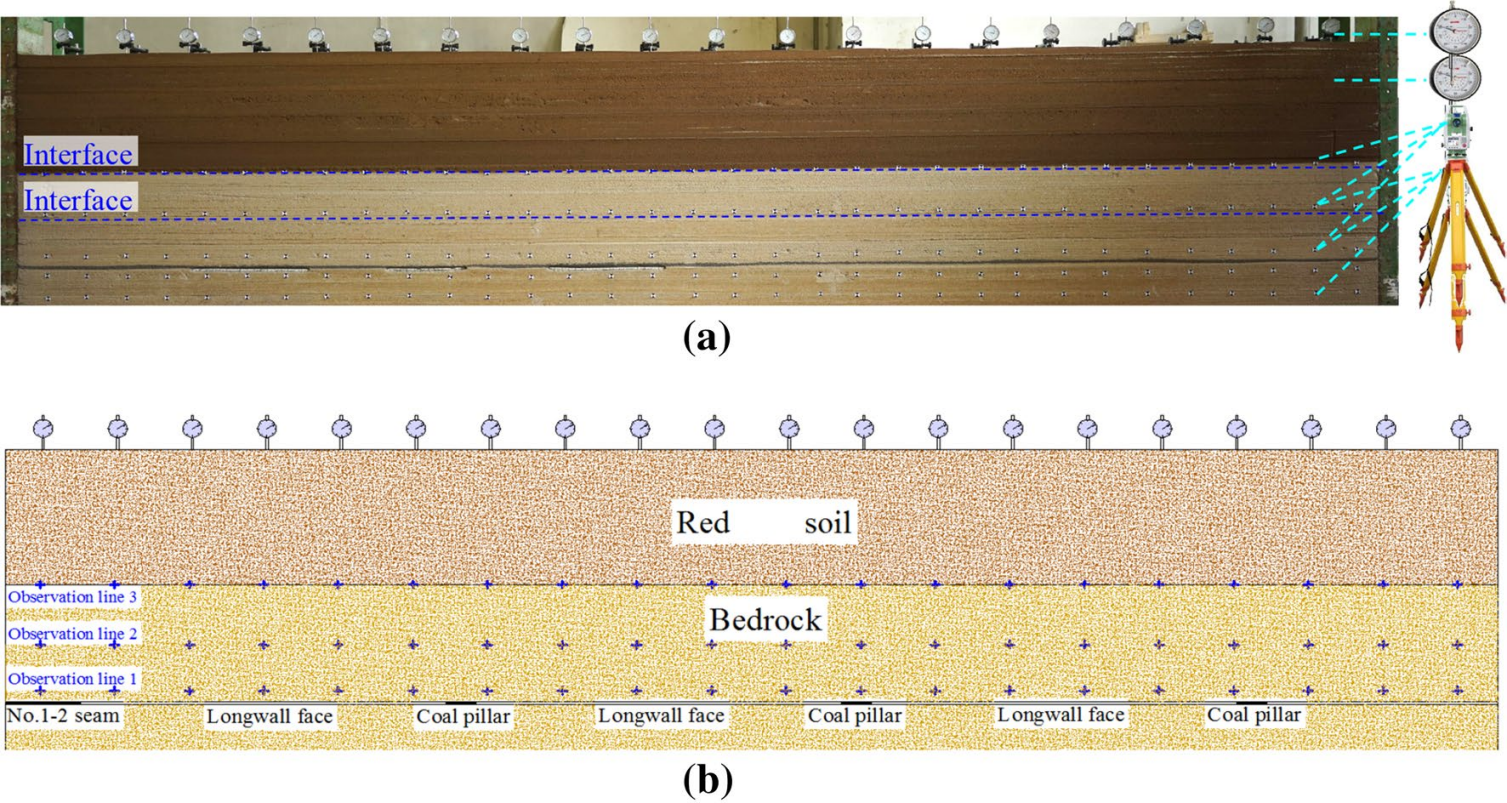
Physical simulation model: (**a**) Simulation model; (**b**) Position of observation lines.

In order to reveal the overburden strata and ground surface subsidence of No. 1-2 seam mining, the physical simulation model was built with the following dimensions: 5 m long × 0.2 m wide × 1.35 m high, the geometric ratio is 1:200 (Fig. 2). During model set up, the simulation materials of bedrock are composed of sand (aggregate), gypsum and calcium carbonate (cementitious materials), as for red soil, the simulation materials and its ratio can be determined by Reference^41^.

The width of coal pillar between faces is 20 m, while the width of boundary pillar is 50 m. Three observation lines were established, and electronic total station was used to monitor the overburden strata subsidence, dial indicators were applied to monitor the ground surface subsidence, the detail position of observation lines is listed below:

- Line 1: 4 cm from the roof of No. 1-2 seam, monitoring the immediate roof subsidence.
- Line 2: 20 cm from the roof of No. 1-2 seam, monitoring the key stratum subsidence.
- Line 3: 40 cm from the roof of No. 1-2 seam, monitoring the interface subsidence.

After No. 1-2 seam mining, based on the subsidence and movement characteristics of the overburden strata, it can be divided into the following three zones in dip direction:

(1) Boundary pillar F-shape zone (BPZ)

**Figure 3:**
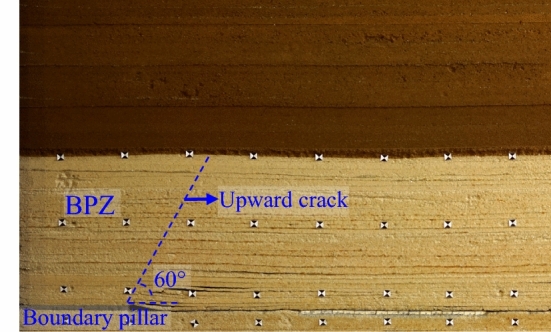
Strata subsidence in BPZ.

The experiment shows that the upward crack is mainly located at the mining boundary, and the development angle is 60° (Fig. 3). Overall, the rock strata above BPZ are unbroken, but due to the rotation of rock strata on the mining side and the load of overburden, rock strata near the upward crack represent deflection.

(2) Trapezoid goaf zone (TGZ)

**Figure 4:**
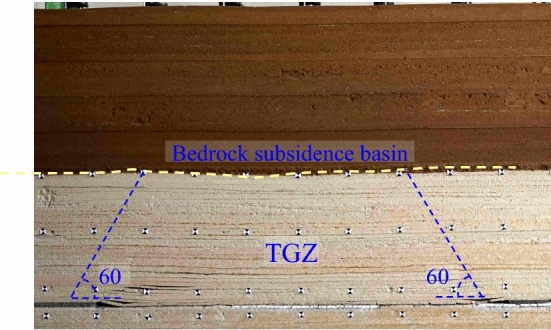
Strata subsidence in TGZ.

The upward cracks develop along the both sides boundaries of goaf, goaf represents trapezoid. The immediate roof caves and fills the goaf, the key stratum in bedrock can form articulated structure, which plays a skeleton role in the subsidence of overburden strata, the subsidence of ground surface depends on the subsidence basin of interface (bedrock subsidence basin). Overall, the subsidence in the middle of goaf is the largest, while it becomes smaller near the upward cracks (Fig. 4).

(3) Coal pillar inverted trapezoid zone (CPZ)

**Figure 5:**
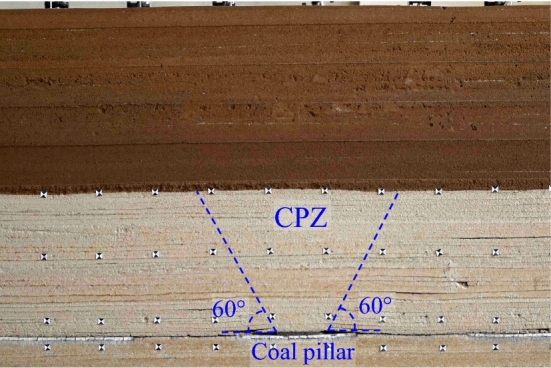
Strata subsidence in CPZ.

The overburden in CPZ represents "inverted trapezoid", Due to the "inverted trapezoid" structure by coal pillar, the subsidence of overburden strata and surface in CPZ is smaller. It can be known that the coal pillar causes the uneven subsidence of ground surface (Fig. 5).

**Figure 6:**
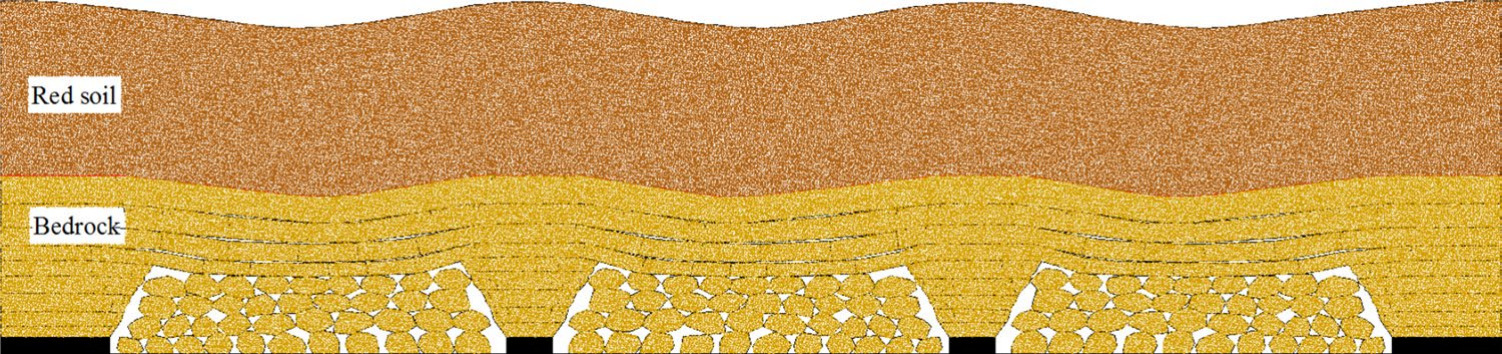
Movement and subsidence of overburden strata in dip direction.

According to physical similarity simulation, the movement and subsidence of overburden strata in dip direction is shown in Fig. 6. The subsidence in TGZ is the largest, while it is smaller in BPZ and CPZ, the movement of soil layer depends on the bedrock subsidence basin. In order to realize the quantitative analysis of the subsidence, it is necessary to establish the subsidence model based on the three zones above, and determine the subsidence of overburden strata and ground surface.

## Subsidence prediction models and subsidence equations

### Subsidence prediction model

**Figure 7:**
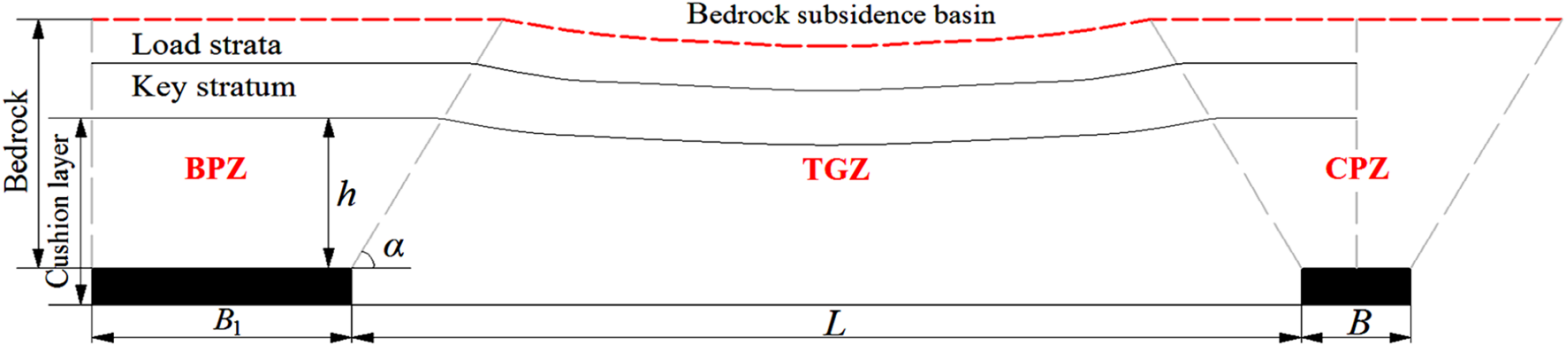
Subsidence prediction model in dip direction.

During mining, the overburden strata caves and moves from bottom to top, the subsidence of soil layer depends on the bedrock, consequently, the bedrock subsidence is analysed firstly. Based on the analysis above, the subsidence prediction model is established and shown in Fig. 7.

Where, *h* is the thickness of strata between the key stratum and coal seam, m; *α* is the caving angle, °; *B*_1_ is width of the boundary pillar, m; *L* is the width of longwall face, m;* B* is width of coal pillar, m. The subsidence equation of the three zones should be solved respectively:

(1) TGZ: The key stratum plays a skeleton role in the bedrock subsidence, and its maximum subsidence value is related to the mining height, thickness of cushion layer and so on. The maximum subsidence is located in the middle of goaf, and it gradually decreases to the both sides, representing symmetrically distribution. The subsidence of load strata depends on the movement of the key stratum, finally it forms “bedrock subsidence basin” at the interface between the bedrock and soil layer.

(2) BPZ: The rock strata under the key stratum and coal seam can be regarded as "cushion layer", the subsidence equation of the key stratum in BPZ can be solved by Winkler elastic foundation beam model^42^. Caving angle had not considered in the in previous studies, in fact, it has an important influence on the subsidence of bedrock and soil layer, consequently it should be considered.

(3) CPZ: The elastic foundation in CPZ is the same as the BPZ, therefore, the improved Winkler elastic foundation beam model also can be used in CPITSZ. There are differences between CPZ and BPZ, one is the range of the zones, and another is that the subsidence curve in CPZ is symmetrical.

### Bedrock subsidence equation

(1) Bedrock subsidence equation of TGZ

**Figure 8:**
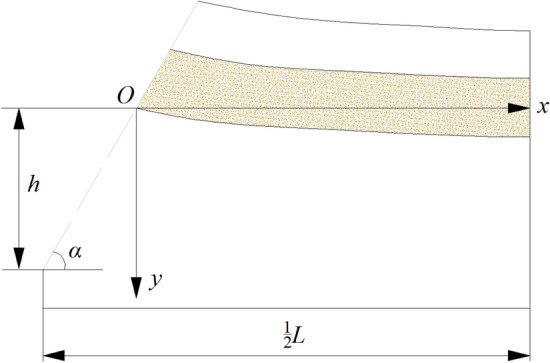
The mechanical model of key stratum in TGZ.

Due to the symmetry of TGZ, half of the zone is taken for study object, the coordinate system is established and shown in Fig. 8, according to subsidence and movement characteristics of the key stratum, the subsidence equation of key stratum in TGZ has been proposed by Reference^12^:

1 $$ y_{1} \left( x \right) = y_{{{\text{max}}}} \left[1 - \frac{1}{{1 + {\text{e}}^{{\left( {x - 0.5l} \right)/a}} }}\right] \quad  \left(0 \le x \le \frac{{L\tan \alpha  - 2h}}{{2\tan \alpha }} \right)$$

where, *y*_1_ is subsidence value of the key stratum in TGZ, m; *l* is the block length of voussoir beam, m; *a* is coefficient related to lumpiness of voussoir beam and coal body stiffness, m, generally it is 0.25* l*; *y*_max_ is the maximum subsidence value of the key stratum, m, it can be determined by the equation below:

2 $$ y_{{{\text{max}}}} = m - h\left( {K_{{\text{p}}} - 1} \right) $$

where, *m* is the mining height, m; *K*_p_ the bulking coefficient of rock between the key stratum and coal seam.

Therefore, the subsidence equation of the key stratum in TGZ is:

3 $$ y_{1} \left( x \right) = \left[ {m - h\left( {K_{{\text{p}}} - 1} \right)} \right] \left[1 - \frac{1}{{1 + {\text{e}}^{{\left( {x - 0.5l} \right)/a}} }}\right] $$

(2) Bedrock subsidence equation of BPZ

**Figure 9:**
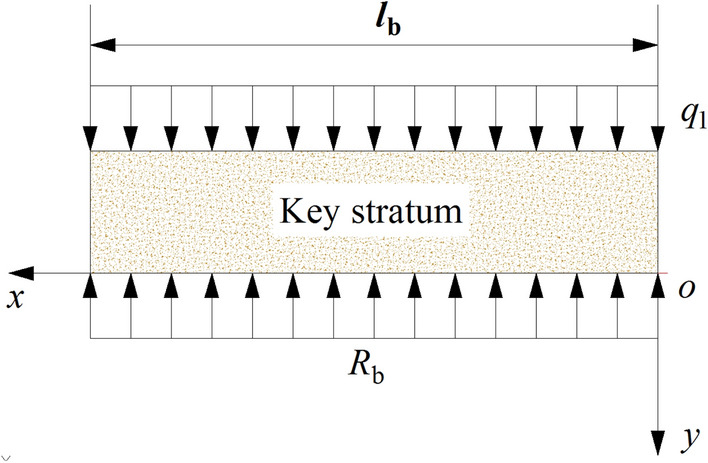
The mechanical model of key stratum in BPZ.

The improved Winkler elastic foundation beam model of the key stratum is established and shown in Fig. 9, *l*_b_ is the length of the key stratum, m; *q*_1_ is the distribution load on the key stratum, MPa; *R*_b_ is the support stress of elastic foundation, MPa, it can be determined as:

4 $$ R_{{\text{b}}} = k_{{\text{b}}} y_{2} $$

5 $$ l_{{\text{b}}} = B_{1} + \frac{h}{\tan \alpha } $$

where, *y*_2_ is subsidence value of the key stratum in BPZ, m; *k*_b_ is the Winkler foundation coefficient, which is related to the thickness and mechanical properties of the elastic foundation, *k*_b_ = *E*_0_/*h*_0_, *E*_0_ is the elastic modulus, MPa; *h*_0_ is the foundation thickness, m.

The unit width of the key stratum is calculated with 1, consequently the subsidence differential equation of the key stratum is:

6 $$ E_{1} I_{1} \frac{{{\text{d}}^{{4}} y_{2} }}{{{\text{d}}x^{4} }} = q_{1} - R_{{\text{b}}} \quad (0  \le x \le l_{b}  )$$

where *E*_1_*I*_1_ is bending rigidity of the key stratum, N·m^2^.

According to Eqs. (4) and (6):

$$ \frac{{{\text{d}}^{{4}} y_{2} }}{{{\text{d}}x^{4} }} + \left( {\frac{{k_{{\text{b}}} }}{{{4}E_{1} I_{1} }}} \right)4y_{2} = \frac{{q_{1} }}{{E_{1} I_{1} }} $$

Let $\beta_{{\text{b}}} = \sqrt[4]{{k_{{\text{b}}} /4E_{1} I_{1} }}$, where, $\beta_{{\text{b}}}$ is characteristic coefficient of the foundation, therefore, the subsidence equation of the key stratum in BPZ is:

$$ y_{2} \left( x \right) = e^{{\beta_{{\text{b}}} x}} \left( {J\cos \beta_{{\text{b}}} x + K\sin \beta_{{\text{b}}} x} \right) + e^{{ - \beta_{{\text{b}}} x}} \left( {U\cos \beta_{{\text{b}}} x + V\sin \beta_{{\text{b}}} x} \right) + \frac{{q_{1} }}{{k_{{\text{b}}} }} $$

When it is far away from the mining boundary, the deflection of the key stratum tends to 0, consequently *J* = *K* = 0, the equation above can be simplified as:

7 $$ y_{2} \left( x \right) = e^{{ - \beta_{{\text{b}}} x}} \left( {U\cos \beta_{{\text{b}}} x + V\sin \beta_{{\text{b}}} x} \right) + \frac{{q_{1} }}{{k_{{\text{b}}} }} $$

According to the relationship between TGZ and BPZ, the boundary conditions of the deflection curve equation are:

8 $$ \left\{ \begin{aligned} &y_{1} \left( {x = 0} \right) = y_{2} \left( {x = 0} \right) \hfill \\& y_{1}^{^{\prime}} \left( {x = 0} \right) = - y_{2}^{^{\prime}} \left( {x = 0} \right) \hfill \\ \end{aligned} \right. $$

According to Eqs. (3), (7) and (8), it can be solved as:

9 $$ \left\{ \begin{aligned} &U = \frac{{y_{{{\text{max}}}} }}{{e^{2} + 1}} - \frac{{q_{1} }}{{k_{{\text{b}}} }} \hfill \\ &V = \frac{{y_{{{\text{max}}}} }}{{e^{2} + 1}} - \frac{{q_{1} }}{{k_{{\text{b}}} }} + \frac{{e^{2} y_{{{\text{max}}}} }}{{a\beta_{{\text{b}}} \left( {e^{2} + 1} \right)^{2} }} \hfill \\ \end{aligned} \right. $$

Therefore, the subsidence equation of the key stratum in BPZ is:

10 $$ y_{2} \left( x \right) = e^{{ - \beta_{{\text{b}}} x}} \left[ {\left( {\frac{{y_{{{\text{max}}}} }}{{e^{2} + 1}} - \frac{{q_{1} }}{{k_{{\text{b}}} }}} \right)\cos \beta_{{\text{b}}} x + \left( {\frac{{y_{{{\text{max}}}} }}{{e^{2} + 1}} - \frac{{q_{1} }}{{k_{{\text{b}}} }} + \frac{{e^{2} y_{{{\text{max}}}} }}{{a\beta_{{\text{b}}} \left( {e^{2} + 1} \right)^{2} }}} \right)\sin \beta_{{\text{b}}} x} \right] + \frac{{q_{1} }}{{k_{{\text{b}}} }} $$

(3) Bedrock subsidence equation of CPZ

**Figure 10:**
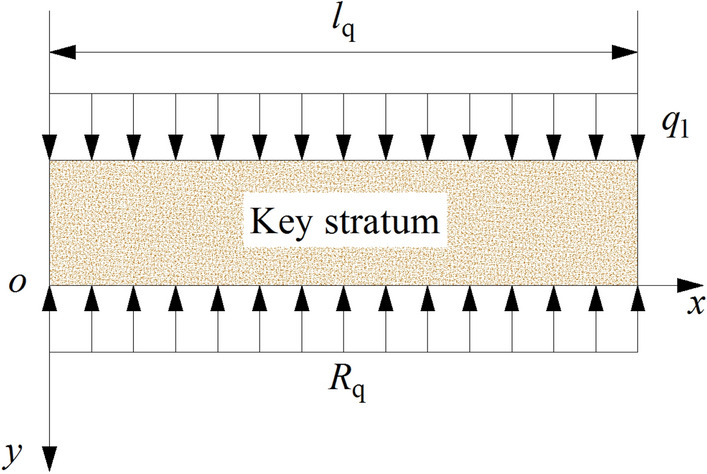
The mechanical model of key stratum in CPZ.

The subsidence curves in CPZ are symmetric, therefore, half of the zone is analysed, the elastic foundation beam model of CPZ is established and shown in Fig. 10. Where, *l*_q_ is the length of half of the key stratum in CPZ, m; *R*_q_ is the support stress of the elastic foundation, MPa, it can be determined as:

11 $$ R_{{\text{q}}} = k_{{\text{q}}} y_{3} $$

12 $$ l_{{\text{q}}} = \frac{B}{2} + \frac{h}{\tan \alpha } $$

where, *y*_3_ is subsidence value of the key stratum in CPZ, m; *k*_q_ is the Winkler foundation coefficient in CPZ, the same as the value in BPZ, $k_{{\text{q}}} = k_{{\text{b}}} = \frac{{E_{0} }}{{h_{0} }}$.

According to the analysis of BPZ, the subsidence curve equation of the key stratum is:

13 $$ y_{3} \left( x \right) = e^{{ - \beta_{{\text{b}}} x}} \left[ {\left( {\frac{{y_{{{\text{max}}}} }}{{e^{2} + 1}} - \frac{{q_{1} }}{{k_{{\text{q}}} }}} \right)\cos \beta_{{\text{b}}} x + \left( {\frac{{y_{{{\text{max}}}} }}{{e^{2} + 1}} - \frac{{q_{1} }}{{k_{{\text{q}}} }} + \frac{{e^{2} y_{{{\text{max}}}} }}{{a\beta_{{\text{b}}} \left( {e^{2} + 1} \right)^{2} }}} \right)\sin \beta_{{\text{b}}} x} \right] + \frac{{q_{1} }}{{k_{{\text{q}}} }} $$

### Soil layer subsidence equation

Due to the soil layer and the bedrock belong to two different medium, therefore, the subsidence characteristics are also different. The subsidence equation of soil layer should be established based on the subsidence equation of bedrock. At present, The stochastic medium theory is most widely used in the subsidence prediction of soil layer, which is recognized by most scholars^7,8,24,25^, According to the probability integration method based on the stochastic medium theory, the subsidence equation of the surface soil layer is:

14 $$ y_{0} (x) = \frac{y\left( x \right)}{2}  \left[ {{\text{erf}}\left( {\frac{\sqrt \pi }{r}x} \right) + 1} \right] $$

where, *y*_0_(*x*) is the subsidence value of surface, m; *r* is the influence radius of soil layer, m; *y*(*x*) is the subsidence value of the “bedrock subsidence basin”, m, it is different in different zones:

15 $$ \left\{ \begin{aligned} &y(x) = y_{1} (x)\quad \text{TGZ} \hfill \\ &y(x) = y_{2} (x)\quad\text{BPZ} \hfill \\ &y(x) = y_{3} (x)\quad\text{CPZ} \hfill \\ \end{aligned} \right. $$

${\text{erf}}\left( {\frac{\sqrt \pi }{r}x} \right)$ is Probability Integral Function, it can be determined by the Probability Integral Table.

According to the analysis above, subsidence of soil layer can be determined.

## Subsidence effect factors of bedrock

### Effect factors of bedrock in TGZ

According to the analysis above, subsidence of soil layer is based on the bedrock subsidence, therefore, the effect factors of bedrock subsidence are mainly analysed. According to the Eq. (3), the bedrock subsidence is mainly related to the mining height *m*, the thickness of rock between the key stratum and coal seam *h*, the bulking coefficient of rock *K*_p_ and the block length of voussoir beam *l*. According to the mining conditions of No. 1-2 seam in Ningtiaota coal mine, the calculation parameters are listed as follows: *l* = 10–16 m, *m* = 1.8–2.5 m, *h* = 6 m, *K*_p_ = 1.15, *L* = 245 m, *α* = 60°.

**Figure 11:**
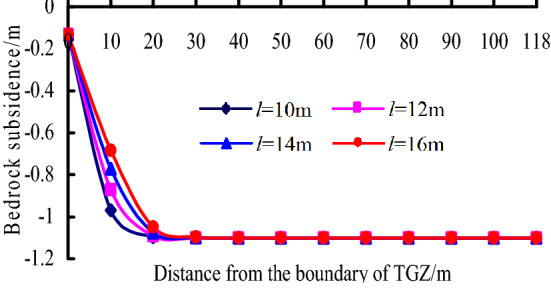
Bedrock subsidence vs. *l* in TGZ.

(1) When the mining height *m* = 2 m and *l* = 10–16 m, the change of bedrock subsidence curves with *l* is shown in Fig. 11. It can be known that:

a. The subsidence value of boundary point is invariable with *l*.

b. Within 30 m from the boundary of TGZ, As *l* increases, the bedrock subsidence acts as unobvious decrease.

c. When it is 30 m away from boundary, bedrock basically reaches fully subsidence, and the subsidence value does not change with *l*.

**Figure 12:**
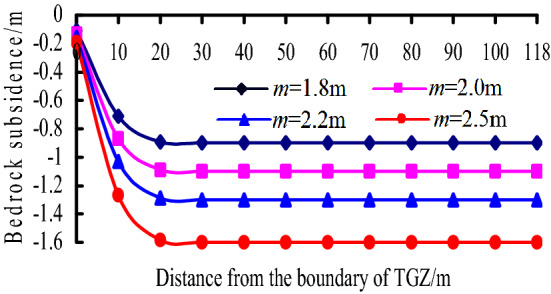
Bedrock subsidence vs. *m* in TGZ.

(2) When the block length of voussoir beam *l* = 12 m and *m* = 1.8–2.5 m, the change of bedrock subsidence curves with *m* is shown in Fig. 12. It can be known that:

a. As *m* increases, subsidence value of boundary point represents unobvious decrease.

b. As *m* increases, the maximum subsidence value decreases obviously, when it is 30 m away from boundary, the bedrock subsidence curves reach the peak value.

Overall, comparing with the block length of voussoir beam *l*, the mining height *m* has more obvious effect on the bedrock subsidence in TGZ.

### Effect factors of bedrock in BPZ

Due to the subsidence equations of BPZ and CPZ are the same, only the range of the two zones is different, therefore, the effect factors of bedrock subsidence in BPZ are studied. According to Eq. (10), the bedrock subsidence in BPZ is mainly related to *m*, *h*, *K*_p_, *E*_0_, *E*_1_*I*_1_, and *q*_1_, based on the mining conditions, the calculation parameters are listed as follows: *E*_0_ = 2500 MPa, *h* = 6 m, *h*_0_ = 8 m, *E*_1_*I*_1_ = 164.6 GN·m^2^, *l* = 12 m, *K*_p_ = 1.15, *α* = 60°, *B* = 20 m, the effect of *m* and *q*_1_ on the bedrock subsidence is analysed.

**Figure 13:**
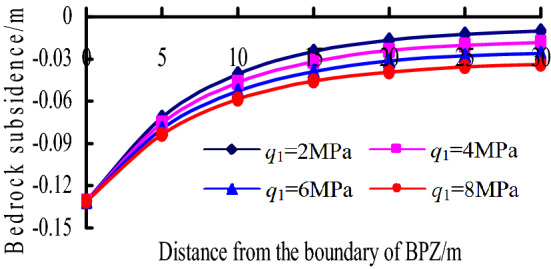
Bedrock subsidence vs. *q*igure in BPZ.

(1) When the mining height *m* = 2 m and *q*_1_ = 2–8 MPa, the change of bedrock subsidence curves with *q*_1_ is shown in Fig. 13. It can be known that:

a. As the distance from the boundary of BPZ increases, the bedrock subsidence decreases at a decreasing speed.

b. With the value of *q*_1_ increases, the bedrock subsidence increases, the subsidence curve is more gentle.

**Figure 14:**
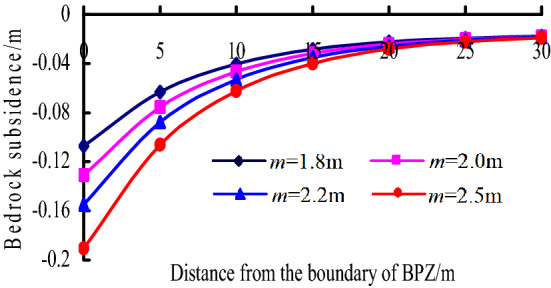
Bedrock subsidence vs. *m* in BPZ.

(2) When the distribution load on the key stratum *q*_1_ = 4 MPa and *m* = 1.8–2.5 m, the change of bedrock subsidence curves with *m* is shown in Fig. 14. It can be known that:

a. Within 15 m from the boundary of BPZ, as the mining height increases, the bedrock subsidence increases obviously, and the closer to the boundary, the faster the bedrock subsidence increases.

b. When it is 15 m away from boundary, the effect of mining height on the bedrock subsidence is unobvious, and the bedrock subsidence value approaches 0.

Overall, the bedrock subsidence in BPZ is mainly related to the mining height and the distribution load on the key stratum. The mining height determines the subsidence value of boundary point, while the distribution load on the key stratum mainly affects the decreasing amplitude of the bedrock subsidence curves.

## Verification of subsidence model

### Theoretical model and physical simulation

Based on the mining conditions of No. 1-2 seam in Ningtiaota coal mine, *m* = 2.0 m, *l* = 12 m, *h* = 6 m, *K*_p_ = 1.15, *L* = 245 m, *α* = 60°, *E*_0_ = 2500 MPa, *h*_0_ = 8 m, *E*_1_*I*_1_ = 164.6 GN·m^2^, *B* = 20 m, *q*_1_ = 4 MPa. According to Eqs. (3), (10) and (13), the bedrock subsidence curve can be obtained by theoretical model calculation.

**Figure 15:**
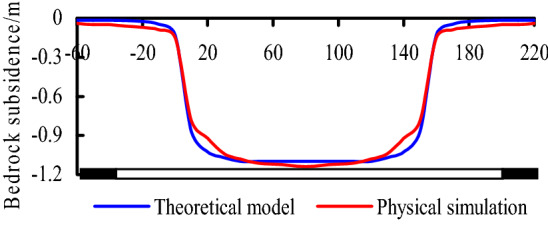
Bedrock subsidence curves.

In addition, according to observation data by physical simulation, the bedrock subsidence curve also can be obtained, the curves were shown in Fig. 15, it can be known that the result of theoretical calculation is basically consistent with physical simulation.

**Figure 16:**
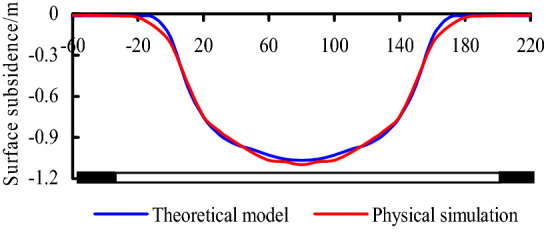
Surface subsidence curves.

The buried depth of No 1-2 seam is 176.6 m (*H* = 176.6 m), and the tangent value of main influence angle tan*β* = 1.8, consequently the influence radius of soil layer *r* = *H*/tan*β* = 98.1 m, combining with Eq. (13), the soil subsidence curve is obtained. Combining with the observation data by physical simulation, the surface subsidence curves are shown in Fig. 16, it can be known that the result of theoretical calculation is basically consistent with physical simulation.

### In-situ subsidence observation of surface

(1) Observation design

**Figure 17:**
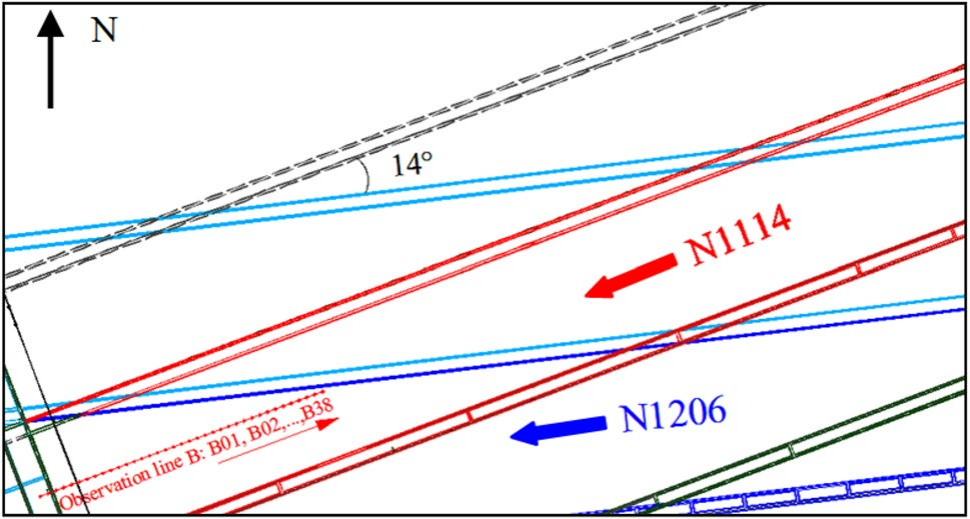
The arrangement of the observation line B.

In-situ observation was carried out to obtain the surface subsidence of No. 1-2 seam mining in Ningtiaota coal mine^43^, the depth of longwall face N1114 is 64–165 m, the thickness of bedrock is 54–66 m, the thickness of soil layer is 10–90 m, and its mining height is 1.9 m. The observation line B is arranged in the middle of the longwall face N1114 (Fig. 17), and it contains 38 observation points (B01, B02,…, B38).

(2) Observation results

**Figure 18:**
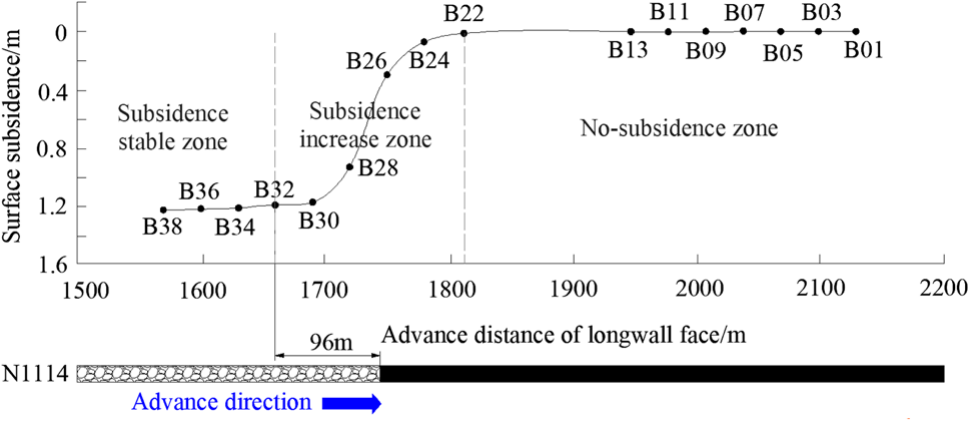
Surface subsidence by in-situ observation.

When N1114 advances 1760 m, the surface subsidence is shown in Fig. 18. It can be divided into three zones, the first is no-subsidence zone which is in front of the longwall face. The second is subsidence increase zone, affected by mining, from Point B22 to B32, the surface subsidence increases. The third is subsidence stable zone, after longwall face advances, the caving roof becomes stable, and the subsidence tends to invariable.

Based on the observation data, the maximum subsidence value affected by N1114 mining is about 1.2 m. According to theoretical model calculation and physical simulation, the maximum subsidence is 1.07 m and 1.1 m respectively, consequently, the results obtained by the three methods are basically consistent.

## Conclusions

During shallow coal seam mining, based on the subsidence characteristics of overburden strata, it can be divided into three structure zones in dip direction: boundary pillar F-shape zone (BPZ), trapezoid goaf zone (TGZ) and coal pillar inverted trapezoidal zone (CPZ). The subsidence in TGZ is the largest, while it is smaller in BPZ and CPZ.

The key stratum has control effect on the subsidence of overburden strata, and the subsidence of soil layer depends on the bedrock subsidence basin. The subsidence mechanical models of the three zones were established, and the subsidence equations of key stratum were given. According to the probability integration method based on the stochastic medium theory, the subsidence equation of the soil layer was obtained.

The effect factors of bedrock subsidence were analysed, the bedrock subsidence is mainly related to the mining height in TGZ, while they are mainly related to the mining height and the distribution load on the key stratum in BPZ and CPZ. the mining height determines the subsidence value of boundary point, while the distribution load on the key stratum mainly affects the decreasing amplitude of the bedrock subsidence curves.

The subsidence curves of bedrock and ground surface were obtained by theoretical model and physical simulation. The maximum subsidence of surface are 1.07 m and 1.1 m respectively by the two methods above, according to in-situ observation, it is about 1.2 m, they are basically consistent.

## Data Availability

The experimental data used to support the findings of this study are included within the article.
